# Crossed Pathways: Tobacco–Cannabis Co‐Use and Motivation to Quit in Young Adults in France

**DOI:** 10.1111/dar.70195

**Published:** 2026-06-25

**Authors:** Tangui Barré, Géraldine Cazorla, Vincent Leroy, Gwenaëlle Maradan, Clotilde Couderc, Julia de Ternay, Carlotta Magnani, Patrizia Carrieri

**Affiliations:** ^1^ Aix Marseille Univ, Inserm, IRD, SESSTIM, Sciences Economiques & Sociales de la Santé & Traitement de l'Information Médicale, ISSPAM Marseille France; ^2^ ORS PACA, Southeastern Health Regional Observatory Marseille France; ^3^ Association Addictions France Paris France; ^4^ Service Universitaire d'Addictologie de Lyon, Hôpital Édouard Herriot, Hospices Civils de Lyon Lyon France; ^5^ Research on Healthcare Performance RESHAPE, INSERM U1290 Villeurbanne France

**Keywords:** cannabis, co‐use, readiness to quit, structural equation model, tobacco

## Abstract

**Introduction:**

Among young adults, intertwined tobacco and cannabis use is a major concern, being linked to poorer cessation outcomes than exclusive use. However, little is known about the shared and simultaneous determinants of motivation to quit both substances, and how the use of one influences readiness to quit the other. We aimed to examine these relationships, focusing particularly on the role of low socioeconomic status.

**Methods:**

In a cross‐sectional survey of French young adults (18–30 years) who co‐use tobacco and cannabis, we modelled pathways to readiness to quit both substances using generalised structural equation modelling (GSEM). Factors associated with four or more lifetime quit attempts for tobacco and cannabis were also assessed using separate binary logistic regressions.

**Results:**

Among 357 participants (54.9% male), GSEM analysis revealed that low socioeconomic status was positively associated with frequency of use (*β* = 0.12, 95% confidence interval [0.04; 0.19] for tobacco; 0.09 [0.02; 0.17] for cannabis) and directly linked to greater readiness to quit both substances (*β* = 0.14 [0.04; 0.24] for tobacco; 0.13 [0.03; 0.23] for cannabis). Further regression analyses suggested that financial strain was the primary driver of these two latter associations with readiness to quit. For both substances, ≥ 4 lifetime quit attempts were associated with material deprivation (adjusted odds ratio 1.39 [1.22; 1.58] for tobacco; 1.24 [1.10; 1.40] for cannabis).

**Discussion and Conclusions:**

In young adults who co‐use tobacco and cannabis, financial strain emerged as a common determinant of motivation to quit, supporting the potential utility of monetary incentives in dual cessation. Further research, including qualitative studies, is needed to clarify cross‐substance effects.

## Key Points

- People who co‐use tobacco and cannabis show poorer cessation outcomes.
- A low socioeconomic status is associated with use frequency in young adults who co‐use.
- Financial strain is directly associated with readiness to quit both substances.
- Higher cannabis use frequency may foster readiness to quit tobacco.
- Readiness to quit cannabis may rise with cannabis dependence.

## 1 Introduction

Tobacco use remains one of the leading global risk factors for premature mortality [1]. Preventing initiation and promoting cessation of tobacco use are therefore public health priorities, especially in Western countries, where a high proportion of all‐cause mortality is attributable to tobacco smoking [1, 2]. Although a downward trend has been observed over the past decade [3], Europe remains one of the regions with the highest smoking prevalence globally [1]. For instance, in 2022, 31.8% of French adults reported smoking tobacco [4].

Young adulthood represents a critical developmental period and a window of heightened vulnerability to psychiatric disorders [5]. Life transitions during this stage provide multiple opportunities for the adoption and reinforcement of smoking as a stable and persistent habit [6, 7]. Therefore, tobacco cessation efforts during young adulthood are particularly critical [7, 8] and may yield greater benefits in terms of life‐years gained [9, 10]. Cannabis co‐use appears to be a key risk factor for the progression to sustained and frequent tobacco use during young adulthood [7, 11, 12].

Generally speaking, cannabis use is associated with increased risk of nicotine dependence and higher frequency of tobacco smoking [12, 13, 14, 15, 16]. Cannabis use is also associated with poorer tobacco cessation outcomes [17, 18, 19, 20, 21], while exposure to nicotine is a risk factor for lapse in cannabis cessation attempt [22]. Individuals who co‐use tobacco and cannabis therefore constitute a particularly relevant target for efforts aimed at reducing tobacco‐related harms [23]. However, to date, while tailoring treatment goals to individuals' interest in quitting may be of potential interest [24], little is known about how the use of one substance influences motivation to quit another (or vice versa) [25, 26]. For example, in a study of young adults in the UK, Walsh et al. recently found no significant correlation between motivations to quit tobacco and cannabis [27].

Cannabis use itself may lead to cannabis use disorder [28], cognitive and academic impairments [29, 30] and mental health problems [31]. It is also associated with respiratory harms and major adverse cardiovascular events [32, 33]. At the European level, recent trends indicate increasing levels of prevalence of use and treatment need, which parallel the increasing proportion of tetrahydrocannabinol found in consumed cannabis [34, 35]. In Europe, including France, cannabis use prevalence is particularly high among young adults, similar to the situation in the US [36, 37, 38]. Since individuals who use cannabis are also likely to use tobacco [39, 40], young adults represent a high‐risk population for tobacco–cannabis co‐use. In 2021, 8.3% of adults aged 18–30 years co‐used in France [41]. For this group, cessation efforts could yield substantial benefits but may also prove particularly challenging. So far, the role of cannabis co‐use when identifying predictors of smoking cessation in young people has not received much attention [42, 43].

Both tobacco [44, 45, 46] and cannabis [36, 47, 48] regular use are more prevalent among people with a low socioeconomic status (SES). People with a low SES are also more likely to experience stronger nicotine [49] and cannabis [50] dependence, which are obstacles to cessation. Conversely, financial strain may enhance motivation to quit smoking, as shown for tobacco in France [51].

As in the general population [52], the number of prior quit attempts has been associated with motivation to quit and tobacco smoking cessation in young adults [53, 54]. In a quasi‐experimental study on a tobacco program in adults, it has been shown that people who tried to quit tobacco four or more times in the past were 2.6 times more likely to quit than those who tried fewer times [55]. Similarly, a greater reported readiness to quit tobacco has been associated with better odds of tobacco cessation in both adults [56] and young adults [57]. We may expect readiness to quit and prior cessation attempts to be also pertinent for assessing motivation and likelihood of future cannabis smoking cessation [58, 59]. To our knowledge, pathways that connect both substance‐use frequencies, SES and such markers of motivation to quit have not been simultaneously studied in people who co‐use. In the UK study previously cited, the authors found no significant association between the frequency of tobacco or cannabis use and quit attempts for either substance [27]. However, the potential influence of SES was not examined in this analysis.

We therefore aimed to identify factors associated with both readiness to quit tobacco and cannabis, and prior quit attempts in young adults who co‐use tobacco and cannabis. More specifically, our objectives were: (i) to develop a model showing how low SES influences levels of readiness to quit, while also examining how tobacco and cannabis use patterns may interact to affect those levels; and (ii) to identify socioeconomic and substance‐use factors associated with a high number of previous quit attempts for both substances.

## 2 Methods

### 2.1 Design

The TOBASCO (TOBAcco‐cannabiS CO‐use in young adults) study was based on a national online cross‐sectional survey conducted between 1 July 2024 and 1 May 2025, in France. The survey was disseminated through multiple channels: (i) social media groups and pages related to addictive behaviours; (ii) social media accounts of cannabis‐related magazines; (iii) mailing lists and social media pages of student organisations; (iv) social media, flyers and posters in local youth support centres (*missions locales*); (v) flyers and posters in student health services; and (vi) the research team's personal network. Flyers and posters were also made available in 16 addiction prevention and treatment centres. Finally, to reach the targeted sample size, a service provider conducted 1 day of in‐person canvassing in a public space (Paris, April 2025).

The TOBASCO study was designed in accordance with the Declaration of Helsinki and received approval from the Aix‐Marseille University ethics committee (IRB00014113, 2023‐12‐14‐01, 14 December 2023). Before accessing the survey questionnaire, participants were required to provide informed consent to participate and to authorise the processing of their personal data.

### 2.2 Participants

TOBASCO targeted individuals aged 18 to 30 who had smoked both tobacco and cannabis within the past 7 days. Co‐use eligibility was assessed at the start of the survey through two separate questions, one for each substance: ‘Have you smoked [tobacco/cannabis] in the past 7 days (either pure or mixed)?’ (Yes/No). Only participants who completed the entire questionnaire were included in the analyses.

### 2.3 Data Collection and Variables Definitions

The average time required to complete the questionnaire was 16 min. Participants were required to answer the questions in the order they appeared in order to proceed through the questionnaire. As a result, there were no missing data. For data privacy reasons, IP addresses were not collected. Consequently, no check for duplicate responses was performed.

Socioeconomic and demographic data collected included gender, age, geographical region, city size, highest degree obtained, job situation (Having a job/Looking for a job/Not having and not looking for a job) and material deprivation. The latter was assessed using six dichotomous (Yes/No) statements [60, 61] which could be summed to obtain a 0–6 composite variable. Self‐perceived household economic status was also evaluated with the following question: ‘Presently, would you say that, financially speaking …’ with six response options from: ‘You are very comfortable’ to ‘You can't manage without going into debt’.

Pattern of substance‐use data included days of use per month (tobacco and cannabis separately), Fagerström Test for Nicotine Dependence [62] (FTND, assessed for participants who reported ≥ 25 days of tobacco use per month; otherwise, the absence of dependence was hypothesised), and the 3‐item Cannabis Use Disorder Identification Test‐Short Form (CUDIT‐SF) [63]. For homogeneity, we will use ‘cannabis dependence’ instead of cannabis use disorder throughout the analyses. The decision to administer the FTND questionnaire only to participants smoking at least 25 days per month was made to minimise questionnaire completion time. This choice is justified by the fact that intermittent smokers have a low likelihood of nicotine dependence [64] and typically achieve low FTND scores [65]. Among light smokers, the FTND provides little additional information beyond the number of cigarettes smoked per day [66]. Indeed, the FTND was originally developed using a sample of individuals smoking an average of one pack per day [62], and its questions are therefore tailored to daily smokers. One question assessed whether cannabis, when smoked, was mixed with tobacco.

Tobacco use frequency was considered as a three‐category variable (non‐daily use/< 10 cigarettes per day/≥ 10 cigarettes per day), as was cannabis use frequency (< 10 days per month/10–29 days per month/daily use). Dependence was assessed using cut‐off values of ≥ 4 and ≥ 2 for FTND and CUDIT‐SF, respectively [62, 63].

Readiness to quit tobacco (or cannabis) was assessed through a ruler: ‘How ready are you to quit smoking tobacco (or cannabis) within the next month (1 = Not at all; 10 = 100% ready)?’ [56].

History of cessation attempts was collected through three questions: ‘In your lifetime, how many attempts have you made to quit for at least 48 hours […]?’ (i) tobacco (while continuing to use cannabis); (ii) cannabis (while continuing to use tobacco); (iii) both tobacco and cannabis simultaneously. The answer modalities were: 0/1 or 2 attempts/3 to 5 attempts/6 to 10 attempts/more than 10 attempts. Modalities were averaged and recoded into: 0/1.5/4/8/12.

The number of lifetime quit attempts was then estimated separately for both tobacco and cannabis. To assess the number of tobacco (respectively, cannabis) quit attempts, we summed tobacco‐only (respectively, cannabis‐only) and tobacco–cannabis (i.e., simultaneous) attempts. We then dichotomised those number of attempts variables to mitigate the risk of unstable estimates and poor model convergence, common challenges in regression when sample sizes are modest. In order to adopt a simplified, consistent approach for both substances and to facilitate the interpretation of the results, we chose to use a common threshold to dichotomise these two variables. We chose a ≥ 4 attempts threshold [55]. This cut‐off was chosen as an intermediate value between 6 and 2. The value of 6 corresponds to the estimated average number of tobacco quit attempts expected before successful quitting, based on recalled quit attempts among adult successful quitters [52]. The value of 2 corresponds to the number of previous tobacco quit attempts associated with a 58% higher odds of 30‐day abstinence at 6‐month follow‐up (compared to no attempt) in young adults [53].

### 2.4 Outcomes

The outcomes were, for both substances: (i) readiness to quit, assessed through the 1–10 rulers; and (ii) ≥ 4 lifetime quit attempts.

### 2.5 Conceptual Model for Modelling Readiness to Quit

Readiness to quit is derived from readiness to change, which refers to the degree to which an individual is motivated to change problematic behaviour patterns. It includes initial attitudinal shifts reflecting dissatisfaction with a behaviour and ongoing change efforts [67]. We considered SES as a reflective latent variable that causes variation in education, job status and income [45, 68]. We designed this SES latent construct for the present study.

We made the following hypotheses that we tested using generalised structural equation modelling (GSEM): (i) a lower SES has a direct positive effect on levels of readiness to quit; (ii) a lower SES has an indirect negative effect on levels of readiness to quit through higher levels of use; (iii) a lower SES has an indirect negative effect on levels of readiness to quit through the presence of dependence; (iv) a higher frequency of use of one substance has a positive direct effect on readiness to quit the other.

The complete rationale for such main hypotheses and for the other ones integrated into the GSEM is provided in Appendix S1. Shortly, our first hypothesis posited that financial strain may motivate individuals to quit tobacco and cannabis [51, 69, 70, 71]. Our second hypothesis proposed that lower SES may increase substance‐use frequency and dependence, primarily through two mechanisms: exposure to a pro‐smoking social environment and the use of substances as a stress‐coping strategy [49, 72, 73, 74, 75]. Higher frequency of use reinforces habits, which in turn act as barriers to quitting [76, 77]. Additionally, it increases dependence, which further reduces readiness to quit [78, 79, 80]. Our third, less‐documented hypothesis suggests that co‐use involves substitution effects [39], compensatory effects [81], and competition for shared resources and posits that high‐frequency use of one substance may increase readiness to quit the other.

**FIGURE 1:**
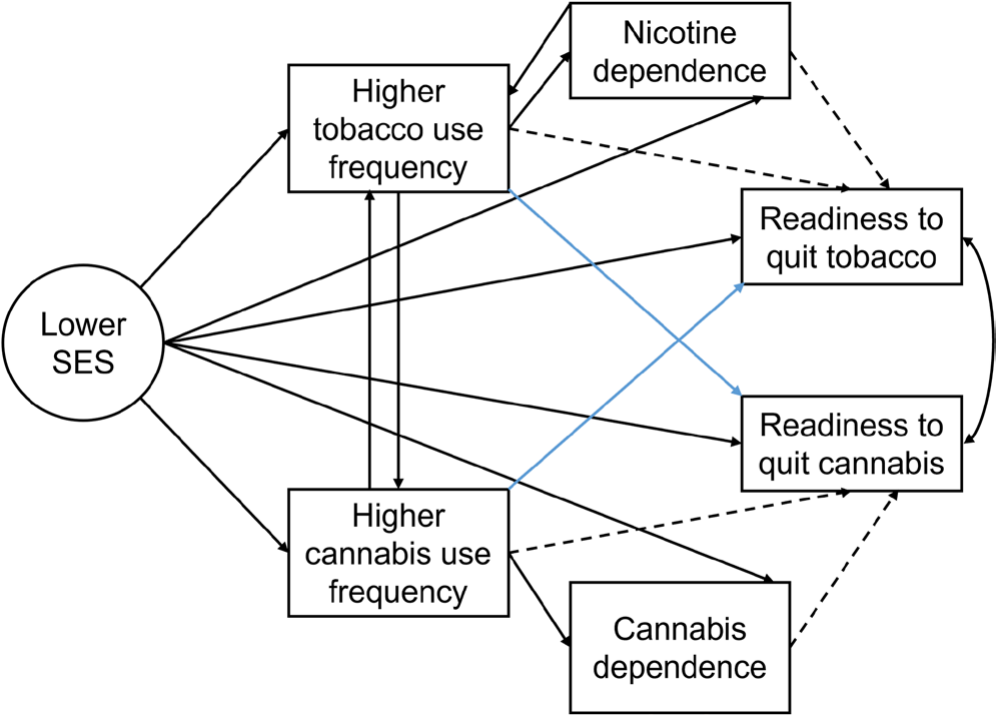
Theoretical pathway relating a lower socioeconomic status to levels of readiness to quit tobacco and cannabis, and their interdependence. SES, socioeconomic status. Plain arrows represent positive effects and dash‐dotted arrows represent negative effects. The double‐headed arrow represents a covariance. Blue arrows are under‐documented and therefore hypothetical. Expected pathways are as follows: A lower SES has direct positive impacts on tobacco and cannabis use frequencies as well as on nicotine and cannabis dependence; a lower SES has a direct positive impact on both levels of readiness to quit; tobacco and cannabis use frequencies have a reciprocal direct positive impact on each other; tobacco use frequency and nicotine dependence have a reciprocal direct positive impact on each other; cannabis use frequency has a direct positive impact on cannabis dependence; tobacco use frequency and nicotine dependence both have a direct negative impact on readiness to quit tobacco; cannabis use frequency and cannabis dependence both have a direct negative impact on readiness to quit cannabis; tobacco use frequency has a direct positive impact on readiness to quit cannabis and cannabis use frequency has a direct positive impact on readiness to quit tobacco; both levels of readiness to quit covary.

Our theoretical pathway to model the impact of SES on both levels of readiness to quit, and their interdependence, is presented in Figure 1. No alternative models to this one have been tested.

### 2.6 Explanatory Variables

#### 2.6.1 Generalised Structural Equation Modelling

The latent construct of SES relied on variables chosen to reflect educational level, job status and income. Educational level ranged from 0 to 8 and corresponded to the highest degree obtained. Job seeking (Yes/No) was used to assess job status. As income was not available, we used measures of financial strain as proxies: material deprivation variables (binary answers to the six previously mentioned statements) and the 4‐category self‐perceived financial situation variable were used as proxies.

Frequency levels of tobacco and cannabis were assessed using the three‐category variables. Dependence variables were binary, age was continuous and gender was assessed as a three‐category variable.

#### 2.6.2 Correlates of Cessation Attempts

Potential correlates of cessation attempts were gender, age, educational level, material deprivation (composite variable ranging from 0 to 6), and frequency levels of tobacco and cannabis use. Except for the composite deprivation variable, the corresponding variables were the same as for the GSEM.

Before using this composite deprivation variable, we checked for its unidimensionality using a principal‐component factor analysis and for its internal consistency using Cronbach's alpha.

### 2.7 Statistical Analyses

#### 2.7.1 Descriptive Statistics

Study sample characteristics were compared according to whether participants had ever attempted to quit tobacco or cannabis, using chi‐square (for categorical variables) and Mann–Whitney (for numerical variables) tests.

#### 2.7.2 Generalised Structural Equation Modelling

We used GSEM (Stata *gsem* command) to test whether our conceptual model (Model 1) (Figure 1) was supported by the TOBASCO data. As, for a given substance, both frequency of use and dependence were: (i) directly related to each other; (ii) expected to be positively influenced by a lower SES; and (iii) expected to negatively impact readiness to quit, we considered deleting dependence variables in an alternative model (Model 2) for parsimony purpose.

Before estimating the structural models, we assessed the measurement model of SES using GSEM, which is equivalent to a confirmatory factor analysis. When the reliability of the latent construct was confirmed, we ran the full models. We expected our sample size to be adapted to our GSEM model, as it was in the upper range of the sample size requirements identified by Wolf et al. for SEM, while presenting only one latent variable [82].

The two full models were fitted using the maximum‐likelihood method assuming logit links for all variables except readiness levels and age (for which identity link and Gaussian distribution were assumed). Levels of readiness to quit were treated as continuous variables [83]. To assess whether this assumption was reasonable, we examined the distribution of responses across the 10 readiness levels, calculated the skewness of both variables and reported the proportion of responses at the extreme values (i.e., 1 and 10). For binary variables, Bernoulli distribution was assumed. Ordinal distribution was assumed for frequency of use and educational level, as well as for self‐perceived financial situation, while multinomial distribution was assumed for gender. Both models were adjusted for age and gender. We computed the total effects of a low SES on readiness levels by summing direct and indirect effects using *nlcom* command. The Akaike information criterion (AIC), the Bayesian information criterion (BIC) and the log‐likelihood values were used in model comparisons, with smaller values indicating a better‐fitting model for AIC and BIC, and greater values indicating better‐fitting model for the log‐likelihood.

Once the best model was chosen, we tested whether the direct effect of SES on levels of readiness to quit could be attributed to financial strain (i.e., deprivation and/or financial situation) by performing linear regression models with readiness levels as outcomes. We separately tested the effects of SES‐related variables: education, job seeking, self‐perceived financial situation, the six deprivation variables and the composite deprivation variable, while adjusting for variables identified on the path from SES to readiness levels, as well as for gender and age.

#### 2.7.3 Correlates of Cessation Attempts

We performed two (one by substance) binary logistic regression models with ‘≥ 4 cessation attempts’ as the outcome. All potential correlates were included regardless of their *p*‐value. Associations were assessed using adjusted odds ratios. We performed sensitivity analyses in which the outcomes were ‘≥ 3 cessation attempts’.

All analyses were performed using Stata software version 17.0 for Windows (StataCorp LP, College Station, TX, USA).

## 3 Results

### 3.1 Study Sample Characteristics

**FIGURE 2:**
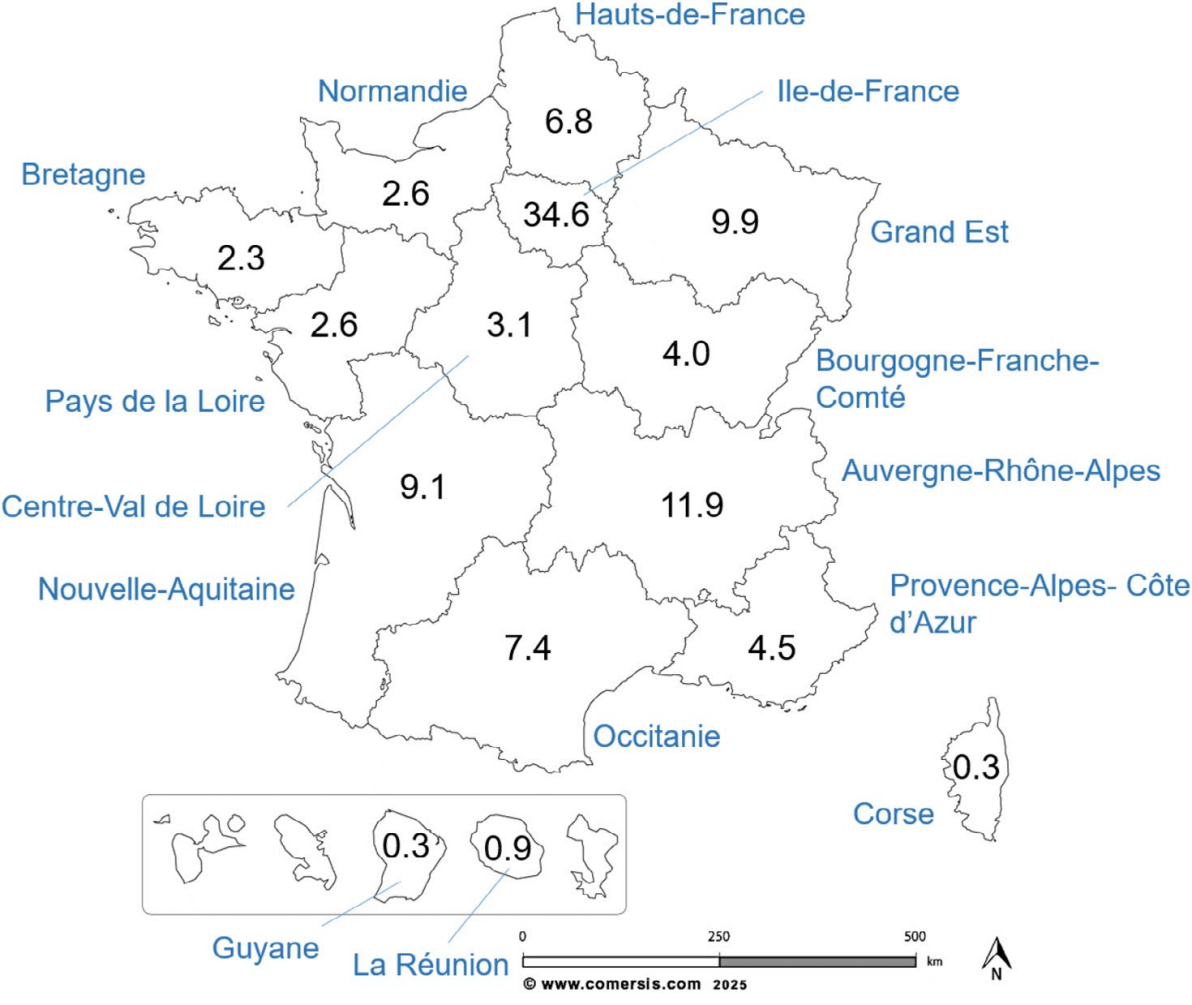
Repartition (in %) of the study population by French region.

From the widespread survey dissemination across multiple channels, a total of 466 people met the eligibility criteria based on age and co‐use questions at the beginning of the questionnaire; of these, 264 (56.7%) completed the survey. In addition, 93 participants were recruited through the in‐person canvassing in a public space, all of whom completed the survey. The study sample therefore comprised 357 participants, spread across France, with 34.6% coming from *Île‐de‐France* (the Paris metropolitan region) (Figure 2). Almost half (47.6%) of participants were recruited through social media, 8.7% via personal invitation, 7.6% through healthcare providers and 36.1% through other channels. More than half (54.9%) of them were men, 41.2% were women and 3.9% chose another answer modality. The median (interquartile range) age was 24 [21; 27] years (Table 1). The majority (87.4%) of the participants always mixed cannabis with tobacco when smoking cannabis.

**TABLE 1:** Study sample characteristics according to lifetime tobacco and cannabis quit attempts (*n* = 357).

	Whole study sample, *n* (%)	< 4 lifetime tobacco quit attempts, *n* (%)	≥ 4 lifetime tobacco quit attempts, *n* (%)	*p*‐value^a^	< 4 lifetime cannabis quit attempts, *n* (%)	≥ 4 lifetime cannabis quit attempts, *n* (%)	*p*‐value^a^
Age, in years, median [IQR]	24 [21; 27]	23 [21; 26]	24 [22; 28]	0.031	23 [21; 26]	24 [22; 27]	0.011
Gender				0.042			0.225
Men	196 (54.9)	119 (50.6)	77 (63.1)		113 (51.6)	83 (60.1)	
Women	147 (41.2)	104 (44.3)	43 (35.2)		98 (44.7)	49 (35.5)	
Other/do not want to answer	14 (3.9)	12 (5.1)	2 (1.6)		8 (3.7)	6 (4.3)	
City size				0.125			0.016
Large city (200,000 inhabitants or more)	180 (50.4)	126 (53.6)	54 (44.3)		123 (56.2)	57 (41.3)	
Medium‐sized city/town	92 (25.8)	53 (22.6)	39 (32.0)		47 (21.5)	45 (32.6)	
Rural area	85 (23.8)	56 (23.8)	29 (23.8)		49 (22.4)	36 (26.1)	
Educational level ≥ upper secondary school certificate^b^	219 (61.3)	141 (60.0)	78 (63.9)	0.469	138 (63.0)	81 (58.7)	0.415
Job seeking	64 (17.9)	42 (17.9)	22 (18.0)	0.970	36 (16.4)	28 (20.3)	0.356
Self‐perceived financial situation				0.035			0.003
You are rather or really comfortable	70 (19.6)	49 (20.9)	21 (17.2)		46 (21)	24 (17.4)	
You are ok	80 (22.4)	60 (25.5)	20 (16.4)		61 (27.9)	19 (13.8)	
You just get by	104 (29.1)	69 (29.4)	35 (28.7)		60 (27.4)	44 (31.9)	
It's difficult to make ends meet/you can't manage without going into debt	103 (28.9)	57 (24.3)	46 (37.7)		52 (23.7)	51 (37.0)	
Material deprivation							
You often have to go without clothing for financial reasons	79 (22.1)	43 (18.3)	36 (29.5)	0.016	42 (19.2)	37 (26.8)	0.091
You often have to go without trips or holidays for financial reasons	137 (38.4)	78 (33.2)	59 (48.4)	0.005	77 (35.2)	60 (43.5)	0.116
You cannot put money aside at the end of the month	141 (39.5)	81 (34.5)	60 (49.2)	0.007	68 (31.1)	73 (52.9)	< 0.001
You often have to go without cultural outings for financial reasons	86 (24.1)	45 (19.1)	41 (33.6)	0.002	43 (19.6)	43 (31.2)	0.013
You often have to go without going out to bars, restaurants, or nightclubs for financial reasons	102 (28.6)	53 (22.6)	49 (40.2)	< 0.001	45 (20.5)	57 (41.3)	< 0.001
You often have to go without food for financial reasons	46 (12.9)	18 (7.7)	28 (23.0)	< 0.001	21 (9.6)	25 (18.1)	0.019
Composite material deprivation^c^	1 [0; 3]	1 [0; 2]	2 [0; 4]	< 0.001	1 [0; 2]	2 [0; 4]	< 0.001
Tobacco use frequency				0.047			0.018
Non‐daily use	101 (28.3)	58 (24.7)	43 (35.2)		67 (30.6)	34 (24.6)	
< 10 cigarettes per day	123 (34.5)	90 (38.3)	33 (27.0)		83 (37.9)	40 (29.0)	
≥ 10 cigarettes per day	133 (37.3)	87 (37.0)	46 (37.7)		69 (31.5)	64 (46.4)	
Cannabis use frequency				0.602			0.003
< 10 days per month	76 (21.3)	50 (21.3)	26 (21.3)		59 (26.9)	17 (12.3)	
10–29 days per month	96 (26.9)	67 (28.5)	29 (23.8)		58 (26.5)	38 (27.5)	
Daily use	185 (51.8)	118 (50.2)	67 (54.9)		102 (46.6)	83 (60.1)	
Nicotine dependence^d^	113 (31.7)	77 (32.8)	36 (29.5)	0.530	63 (28.8)	50 (36.2)	0.140
Cannabis dependence^e^	272 (76.2)	170 (72.3)	102 (83.6)	0.018	150 (68.5)	122 (88.4)	< 0.001
Readiness to quit tobacco (median [IQR])^f^	5 [3; 8]	5 [3; 7]	7 [5; 9]	< 0.001	5 [3; 7]	6 [4; 8]	0.010
Readiness to quit cannabis (median [IQR])^f^	4 [2; 7]	4 [2; 6]	5 [3; 7]	0.005	4 [2; 6]	5 [3; 8]	< 0.001

Abbreviations: CUDIT‐SF, Cannabis Use Disorder Identification Test‐Short Form; FTND, Fagerström Test for Nicotine Dependence; IQR, interquartile range.

^a^ Chi‐square (for categorical variables) and Mann–Whitney (for numerical variables) tests.

^b^ This 8‐modality variable was dichotomised to ease table readability. Answers included in the lower educational level (i.e., < upper secondary school certificate) were: No lower secondary school diploma; Lower secondary school diploma (*Brevet des collèges*); Vocational diploma (*BEP, CAP, BP*, etc.); Vocational baccalaureate. Answers included in the higher educational level (i.e., ≥ upper secondary school certificate) were: General or technological baccalaureate; Two‐year higher education diploma (*BTS, DUT*, etc.); Three‐ or four‐year higher education degree (*Licence, Maîtrise*, etc.); Master's degree or higher (Master, engineering degree, etc.).

^c^ This variable is the sum of self‐reported deprivations among the six previously mentioned in the table.

^d^ Fagerström Test for Nicotine Dependence ≥ 4 [62].

^e^ Cannabis Use Disorder Identification Test‐Short Form ≥ 2 [63].

^f^ Self‐reported on a scale from 1 (‘Not at all’) to 10 (‘100% ready’).

For readiness to quit tobacco, response proportions across modalities ranged from 4.2% (modality ‘2’) to 14.6% (modality ‘5’), with both extreme values (1 and 10) representing less than 14% and skewness was 0.002. For readiness to quit cannabis, proportions ranged from 3.9% (modality ‘9’) to 17.1% (modality ‘1’), with 7.8% of responses at modality ‘10’ and skewness was 0.467. The median level of readiness to quit tobacco was greater than the median level of readiness to quit cannabis (Wilcoxon signed‐rank test, *p* < 0.001). Around a fifth (22.4%) of the study population had never made any attempt. Median [IQR] numbers of tobacco and cannabis quit attempts were 1.5 [0; 5.5] and 1.5 [0; 8], respectively (Wilcoxon signed‐rank test, *p* = 0.035). Of the 357 participants, 122 (34.2%) and 138 (38.7%) had made ≥ 4 tobacco and cannabis cessation quit attempts, respectively (*p* = 0.121). The respective percentages for ≥ 3 attempts were 43.1% and 49.0% for tobacco and cannabis, respectively (*p* = 0.031).

### 3.2 Measurement Model of SES

Results of the reflective measurement model are provided in Figure S1. All indicators were significantly related to the SES latent variable (*p* < 0.001) in the expected directions, that is, the greater the latent variable was, the lower the SES was. The variance of SES was estimated at 11.31 (95% confidence interval [5.86; 21.83]).

### 3.3 Full Generalised Structural Equation Models

Results of Model 1 are provided in Table S1. The path cannabis dependence → readiness to quit cannabis was positive but did not reach statistical significance (*β* = 0.65 [−0.04; 1.34]), while cannabis use frequency → readiness to quit cannabis was negative and significant.

Therefore, removing cannabis dependence from the model was not pertinent, as cannabis use frequency and dependence had opposite effects on readiness to quit cannabis. Only nicotine dependence was therefore removed in the alternative model.

In Model 2, the only non‐significant paths were: (i) frequency of tobacco use → readiness to quit cannabis (*β* = 0.29 [−0.07; 0.65]); (ii) cannabis dependence → readiness to quit cannabis (*β* = 0.66 [−0.03; 1.35]) (Table S1). The total effect of SES on readiness to quit cannabis was significant (coefficient = 0.16 [0.02; 0.30], *p* = 0.027), but the total effect of SES on readiness to quit tobacco was not (coefficient = 0.07 [−0.05; 0.19], *p* = 0.257).

**FIGURE 3:**
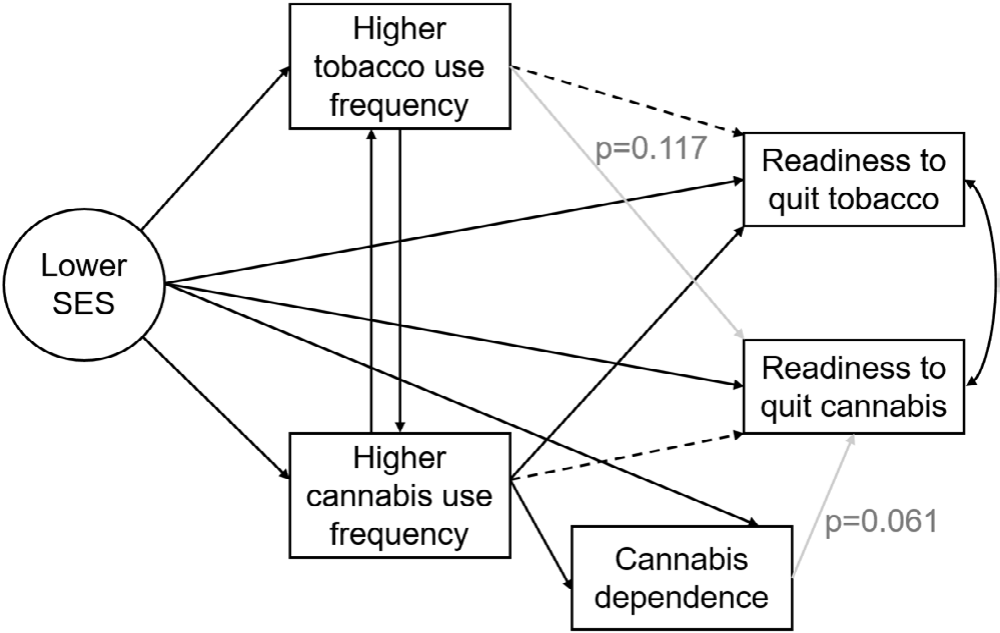
Final pathway relating a lower socioeconomic status to levels of readiness to quit tobacco and cannabis. Variables used to measure a low socioeconomic status (SES) are not represented; neither are age and gender. Plain arrows represent positive effects, and dash‐dotted arrows represent negative effects. The double‐headed arrow represents a covariance. Non‐significant relationships (*p* > 0.05) are represented in grey. A lower SES was positively associated with tobacco and cannabis use frequencies, as well as with cannabis dependence. A lower SES was positively associated with both levels of readiness to quit. Tobacco and cannabis use frequencies were reciprocally positively associated. Cannabis use frequency was positively associated with cannabis dependence. Tobacco use frequency was inversely associated with readiness to quit tobacco, and cannabis use frequency was inversely associated with readiness to quit cannabis. Cannabis dependence was non‐significantly positively associated with readiness to quit cannabis. Tobacco use frequency was non‐significantly positively associated with readiness to quit cannabis. Cannabis use frequency was positively associated with readiness to quit tobacco. Both levels of readiness to quit covaried.

AIC, BIC and log‐likelihood values for the two models are provided in Table S2. The alternative model had better model fit indices than the first model. We therefore kept it (Figure 3).

Unidimensionality of the composite deprivation variable was confirmed, with a first factor explaining 54.6% of the variance and all loadings > 0.6 on this factor. Internal consistency was also confirmed (Cronbach's alpha = 0.828), with all item‐test and item‐rest correlations > 0.5.

Based on the selected model (Figure 3), in linear regression models, when testing separately variables participating in the SES construct, education, job seeking and self‐perceived financial situation were not associated with readiness levels (except for ‘you just get by’ and readiness to quit cannabis). Four material deprivation variables were associated with at least one readiness level (deprivation of trips and of cultural outings were not), and the composite deprivation variable was associated with both levels (Table S3).

### 3.4 Correlates of Cessation Attempts

In binary logistic regression, reporting at least 4 lifetime tobacco cessation attempts was associated with being a man, material deprivation, a higher educational level and non‐daily tobacco use (Table 2). Reporting at least 4 cannabis cessation attempts was associated with material deprivation and ≥ 10 days per month of cannabis use (Table 2). Given the negative association observed between cannabis dependence and readiness to quit cannabis in our GSEM analyses, which, despite not being statistically significant, suggests a potentially meaningful effect with substantial uncertainty due to the wide confidence interval, we conducted a *post hoc* analysis by adding cannabis dependence to the regression model. In this model, the modality ‘10‐29 days per month’ was no longer significant (*p* = 0.196); neither was daily use (*p* = 0.097).

**TABLE 2:** Factors associated with having made at least 4 lifetime cessation attempts (binary logistic regressions, *n* = 357).

	Tobacco		Cannabis		Cannabis (post hoc)	
	aOR [95% CI]	*p*‐value	aOR [95% CI]	*p*‐value	aOR [95% CI]	*p*‐value
Age, in years (median [IQR])	1.05 [0.98; 1.13]	0.200	1.07 [1.00; 1.15]	0.062	1.07 [1.00; 1.15]	0.050
Gender						
Men (ref.)	1		1		1	
Women	0.56 [0.34; 0.93]	0.026	0.73 [0.45; 1.19]	0.211	0.71 [0.43; 1.17]	0.183
Other/do not want to answer	0.17 [0.05; 0.65]	0.009	0.91 [0.25; 3.30]	0.885	0.84 [0.22; 3.20]	0.799
Composite material deprivation^a^	1.39 [1.22; 1.58]	< 0.001	1.24 [1.10; 1.40]	0.001	1.22 [1.08; 1.39]	0.001
Educational level^b^	1.17 [1.01; 1.34]	0.033	1.06 [0.93; 1.21]	0.368	1.08 [0.94; 1.23]	0.278
Tobacco use frequency						
Non‐daily use (ref.)	1		1		1	
< 10 cigarettes per day	0.41 [0.21; 0.78]	0.006	0.73 [0.38; 1.40]	0.345	0.76 [0.39; 1.48]	0.416
≥ 10 cigarettes per day	0.52 [0.28; 0.97]	0.041	1.36 [0.75; 2.45]	0.312	1.45 [0.79; 2.66]	0.230
Cannabis use frequency						
< 10 days per month (ref.)	1		1		1	
10–29 days per month	0.76 [0.37; 1.53]	0.437	2.24 [1.11; 4.50]	0.024	1.63 [0.78; 3.43]	0.196
Daily use	1.33 [0.67; 2.63]	0.417	2.79 [1.40; 5.59]	0.004	1.89 [0.89; 4.00]	0.097
Cannabis dependence^c^	—		—		2.96 [1.54; 5.68]	0.001

Abbreviations: aOR, adjusted odds ratio; CI, confidence interval; IQR, interquartile range.

^a^ This variable, ranging from 0 to 6, corresponds to the sum of self‐reported deprivations.

^b^ This variable, ranging from 0 to 8, corresponded to the highest diploma obtained in the following list: Lower secondary school diploma (*Brevet des collèges*); Vocational diploma (*BEP, CAP, BP*, etc.); Vocational baccalaureate; General or technological baccalaureate; Two‐year higher education diploma (*BTS, DUT*, etc.); Three‐ or four‐year higher education degree (*Licence, Maîtrise*, etc.); Master's degree or higher (Master, engineering degree, etc.).

^c^ Cannabis Use Disorder Identification Test‐Short Form ≥ 2 [63].

When lowering the threshold to at least three attempts, gender and educational level were no longer associated with the tobacco outcome. Material deprivation and 10–29 days of cannabis use per month were no longer associated with the cannabis outcome (Table S4).

## 4 Discussion

In a gender‐balanced sample of young adults in France who co‐use tobacco and cannabis, we found that material deprivation was directly associated with higher readiness to quit both substances and with reporting four or more cessation attempts for each substance. A lower SES was positively associated with readiness to quit cannabis when considering the total path (including direct and indirect effects). Lastly, we highlighted a direct association between higher cannabis use frequency and readiness to quit tobacco.

For both substances, our results suggest that financial strain fosters motivation to quit in young adults who co‐use. Saving money is indeed a commonly reported reason to quit tobacco [79, 80] and cannabis [71, 84] in young adults. Rising tobacco prices are an effective intervention to activate this lever to increase intention to quit and quit attempts, especially in low‐income groups [85, 86, 87]. However, price intervention alone is unlikely to translate in successful cessation in the most deprived individuals [86, 87]. For such populations, implementation of multicomponent interventions including elements such as social support and pharmacotherapy is critical [88].

While the published literature is inconclusive about the type of tobacco intervention that is most effective for young adults [89], we can expect, from our findings, that emphasising money savings when preparing a quit attempt or during it may foster motivation in young adults who co‐use. As a group, people who co‐use tobacco and cannabis are moreover likely to have lower financial resources [41].

This similar association of financial strain with readiness to quit and quit attempts for both substances suggests that financial incentives may be particularly suited to help people who co‐use to quit both simultaneously. Indeed, financial incentives have been shown to increase motivation to quit [90] and long‐term rates of tobacco smoking cessation [91, 92], especially among people with financial needs [93]. Few data are available for cannabis cessation [94, 95], but a recent randomised trial suggests that monetary incentive is effective at reducing cannabis use in people with heavy use [96]. A small pilot study also supported the feasibility and acceptability of monetary incentives in people who co‐use [97].

We found no overall association between low SES and readiness to quit tobacco, which seemingly reflects competing pathways. On the one hand, low SES is directly and positively associated with readiness to quit tobacco (supposedly through financial strain), and indirectly positively associated with it through cannabis use frequency. On the other hand, low SES is positively associated with tobacco use frequency, which is negatively associated with readiness to quit tobacco. The positive pathways appear to be cancelled out by the negative one. Similarly, frequency of use and financial strain showed opposing associations with the likelihood of reporting multiple tobacco quit attempts. A higher educational level was positively associated with having made at least 4 tobacco quit attempts, highlighting that all dimensions of the SES do not have similar effects on quitting motivation outcomes. In this small sample of young adults, our results therefore suggest that individuals with a high tobacco use frequency, a low educational level, but no strong financial strain, may be the least motivated to quit tobacco.

An original perspective that emerged from our cross‐sectional study is that a higher cannabis use frequency seemed to have a positive impact on readiness to quit tobacco. Some hypotheses can be suggested. First, we may expect some people with heavy cannabis use to endorse a cannabis user identity [98, 99, 100, 101], but wanting to get rid of the tobacco user identity which may be less desirable or more stigmatised [102, 103]. Given that cannabis may, to some extent, act as a substitute for tobacco [104, 105, 106, 107, 108], it is also plausible that, for some participants, readiness to quit tobacco led to a reduction in its use, which was subsequently compensated by an increase in cannabis consumption. Drawing on the compensatory health beliefs model [81], we may hypothesise that readiness to quit tobacco could serve as a ‘compensatory intention’, offsetting the perceived negative effects of frequent cannabis use. We may also hypothesise that some people using cannabis frequently employ tobacco solely as a mulling agent (e.g., to ensure proper combustion of cannabis joints [108]) and, as a result, may feel ready to quit tobacco—despite feeling technically constrained in their cannabis use. Finally, although no empirical evidence currently supports this hypothesis, individuals with limited financial resources may prioritise quitting tobacco to allocate more funds to cannabis, particularly if the latter is perceived as offering greater satisfaction, fewer side effects [108, 109], or reduced inconvenience compared to tobacco.

That said, it is important to consider the route of cannabis administration and what young adults mean by ‘tobacco cessation’. In France, as in Europe [40], cannabis is predominantly consumed in joints containing tobacco [110]. Thus, frequent cannabis use generally entails concomitant tobacco use through this route of administration. Quitting tobacco while continuing cannabis use would therefore require adopting a tobacco‐free route of cannabis administration (e.g., smoking pure cannabis, using a vaporizer or consuming edibles). It is also possible that by ‘tobacco cessation’, some participants understood ‘tobacco cigarette cessation’, perceiving their tobacco and cannabis use as disconnected [108], and thus considered quitting cigarettes while continuing to smoke tobacco‐containing joints. Such a situation would imply ongoing exposure to nicotine and could thereby foster relapse after tobacco cessation.

Qualitative studies are needed to shed light on those questions and hypotheses. The reverse association (i.e., a greater tobacco use frequency enhancing readiness to quit cannabis) did not reach statistical significance and should be further explored in studies with a larger sample size. Future findings in this direction might help understand potential superiority of sequential rather than simultaneous quitting in some people [111].

The associations we found suggest that reducing tobacco use may increase both readiness to quit tobacco and the likelihood of making a quit attempt. In contrast, they suggest that reducing cannabis use may decrease readiness to quit tobacco, while showing no significant effect on readiness to quit cannabis and unclear effects on the likelihood of attempting to quit. Those effects need to be confirmed by longitudinal studies, but our observations suggest that among people who co‐use, reducing cannabis use—a commonly observed behaviour [58]—may not constitute a desirable step towards dual cessation, whereas reducing tobacco use may be. It is worth noting that, according to a 2019 Cochrane review [112], neither reduction‐to‐quit nor abrupt tobacco cessation interventions showed superior long‐term quit rates compared with each other. To date, no clear superiority has been demonstrated between simultaneous and sequential dual cessation approaches [111].

In line with what was found elsewhere [113], cannabis dependence (vs. no cannabis dependence) showed a positive (albeit non‐significant) effect on readiness to quit cannabis. This discrepancy in the dependence‐to‐readiness relationships between tobacco and cannabis may partly reflect differences in the assessment tools used. The FTND primarily captures patterns of cigarette use and the inability to refrain from smoking [62] whereas the CUDIT‐SF items emphasise interference with daily functioning and negative consequences of cannabis use [63]. Further studies are needed to test how these two dimensions of substance‐use disorders influence motivation to quit tobacco and cannabis. Another possible explanation is the stronger intoxicating properties of cannabis: its acute and chronic effects on cognitive functioning [114] may encourage cessation because they impede daily functioning, while acute cognitive effects of nicotine tend to be beneficial [115].

In our sample, readiness to quit tobacco was higher than readiness to quit cannabis. This pattern is consistent with previous studies showing greater interest or desire to quit tobacco than cannabis among adults [106], young adults [116], college students [117] and adolescents [118] as well as with baseline findings from Becker et al.'s trial on dual cessation [119]. However, we observed somewhat more quit attempts for cannabis than for tobacco.

To explain this seemingly discrepancy, we may expect people who use cannabis to make many, and often rapid and unprepared, transitions between regular use, reduction and abstinence [58], resulting in a greater number of quit attempts according to our definition. Moreover, we may expect young adults to have lower expectancy of success with quitting tobacco than cannabis [116], resulting in less tobacco quit attempts. Lastly, as previously mentioned, cannabis‐related impairments in daily functioning may prompt spontaneous quit attempts, even without adequate preparation or elevated readiness to quit. By contrast, tobacco use is less likely to trigger such spontaneous quit attempts. Rather, its health‐related adverse effects are typically perceived only in the long term. For instance, older individuals are more likely to be motivated to quit tobacco by health concerns [120]. We can thus hypothesise that tobacco cessation attempts may require more mature forms of motivation, which are less likely to be present in young adults.

Our results are derived from cross‐sectional data, which precludes causal interpretation. Moreover, they rely on hypothesised directional associations that somehow remain questionable. For example, it is plausible that readiness to quit tobacco reduced tobacco use frequency, as tobacco use reduction may be an intermediate stage before cessation [121, 122]. The same may be true for cannabis. This uncertainty is further compounded by the possibility of model equivalence, whereby alternative causal structures cannot be distinguished based on our cross‐sectional data alone. Future longitudinal research is essential to clarify the directional nature of these relationships and validate our proposed model.

The first strength of this study lies in the assessment of different markers of motivation to quit both tobacco and cannabis. This approach makes it possible to examine the putative mechanisms underlying both motivations simultaneously among co‐users, and to compare them in parallel. Focusing on people who co‐use is original and enables us to extend some results found in studies exploring only one substance. Indeed, as tobacco use is frequent in people who use cannabis, especially in Europe [40, 41], we thus took into account the major cofounder, which is tobacco, when studying motivation to quit cannabis.

The models we used were expected to capture the direct and indirect effects of SES on readiness to quit. However, we acknowledge that factors unrelated to SES, which were not included in our models, may also impact readiness to quit, such as cognitive, psychological, or environmental factors. Our study population was gender‐balanced and came from diverse French regions. However, the recruitment process did not lead to a representative sample of young adults who co‐use because of recruitment biases and a slight overrepresentation of people in the Paris region (in 2025, it accounted for 21.6% of the 20–39 year population) [123]. Specifically, our recruitment procedure may have introduced a selection bias by favouring the inclusion of individuals already motivated to quit, while potentially excluding those less willing to disclose their substance use or motivations due to social desirability concerns. Consequently, our study sample may overrepresent individuals with higher quit intentions. Our method did not allow us to reliably assess the response rate. We cannot exclude the possibility that participants who completed the survey differed systematically from those who dropped out before completion, especially as we implemented forced answering. While there is no conclusive pattern of results for the impact of forced answering on data quality, its implementation may have impacted answer quality [124]. Additionally, the questionnaire did not include attention checks or internal reliability checks for responses.

Another limitation is the sample size, which was limiting in testing more complex pathways, such as treating material deprivation or financial strain as a separate latent variable (i.e., as a mediator between low SES and readiness to quit).

Due to the nature of our variables, we had to use GSEM, which, unlike SEM, does not allow for the calculation of goodness‐of‐fit indices such as the Comparative Fit Index or the Root Mean Square Error of Approximation. Readers should bear this limitation in mind when assessing the robustness of our conclusions. Cannabis use frequency was assessed in number of days of use per month, but joints per day, quantities, and cannabis potency were not taken into account, as well as possible other routes of administration, while we know they are important in characterising cannabis use and its effects [125, 126]. By limiting the administration of the FTND to people who smoke at least 25 cigarettes a day, we may have underestimated nicotine dependence. Lastly, tobacco use was assessed in cigarettes per day, but the role of tobacco within joints was not estimated.

## 5 Conclusion

To conclude, financial strain was consistently associated with higher motivation to quit both tobacco and cannabis in young adults who co‐use. Financial levers may thus be particularly pertinent in interventions targeting dual cessation in this population. Qualitative research can now be useful to better explore the putative beneficial impact of a high cannabis use frequency on readiness to quit tobacco.

## Author Contributions

Conceptualization: T.B. Data curation: V.L. Formal analysis: T.B. Funding acquisition: T.B., P.C. Investigation: T.B., G.C., G.M., C.C., J.T., C.M. Project administration: T.B., G.C. Writing – original draft: T.B. Writing – review and editing: All authors.

## Funding

This study received funding from the 2023 call for projects on ‘Psychoactive substances and addictive behaviors’ (AAP‐2023‐SPA‐17, project #23IReSP093), managed by the IReSP (French Institute for Public Health Research) and the INCa (French National Cancer Institute).

## Conflicts of Interest

The authors declare no conflicts of interest.

## Supporting information


**Figure S1:** Measurement model of the socioeconomic construct. The six squares at the bottom represent material deprivation variables.

**Appendix S1:** Rationale for pathway design and generalised structural equation modelling hypotheses.

**Table S1:** Results of the first and alternative models (generalised structural equation modelling, *n* = 357).

**Table S2:** Information criteria and model comparison metrics (generalised structural equation modelling, *n* = 357).

**Table S3:** Associations between socioeconomic‐related variables and levels of readiness to quit tobacco and cannabis (linear regression models, *n* = 357).

**Table S4:** Factors associated with having made at least 3 lifetime cessation attempts (binary logistic regressions, *n* = 357).

## Data Availability

The data that support the findings of this study are available on request from the corresponding author. The data are not publicly available due to privacy or ethical restrictions.
