# Supplementary figures and images for: Crossed Pathways: Tobacco–Cannabis Co‐Use and Motivation to Quit in Young Adults in France
Source: Drug Alcohol Rev. 2026 Jun 25;45(5):e70195. doi: 10.1111/dar.70195 (PMC13305342; doi:10.1111/dar.70195)

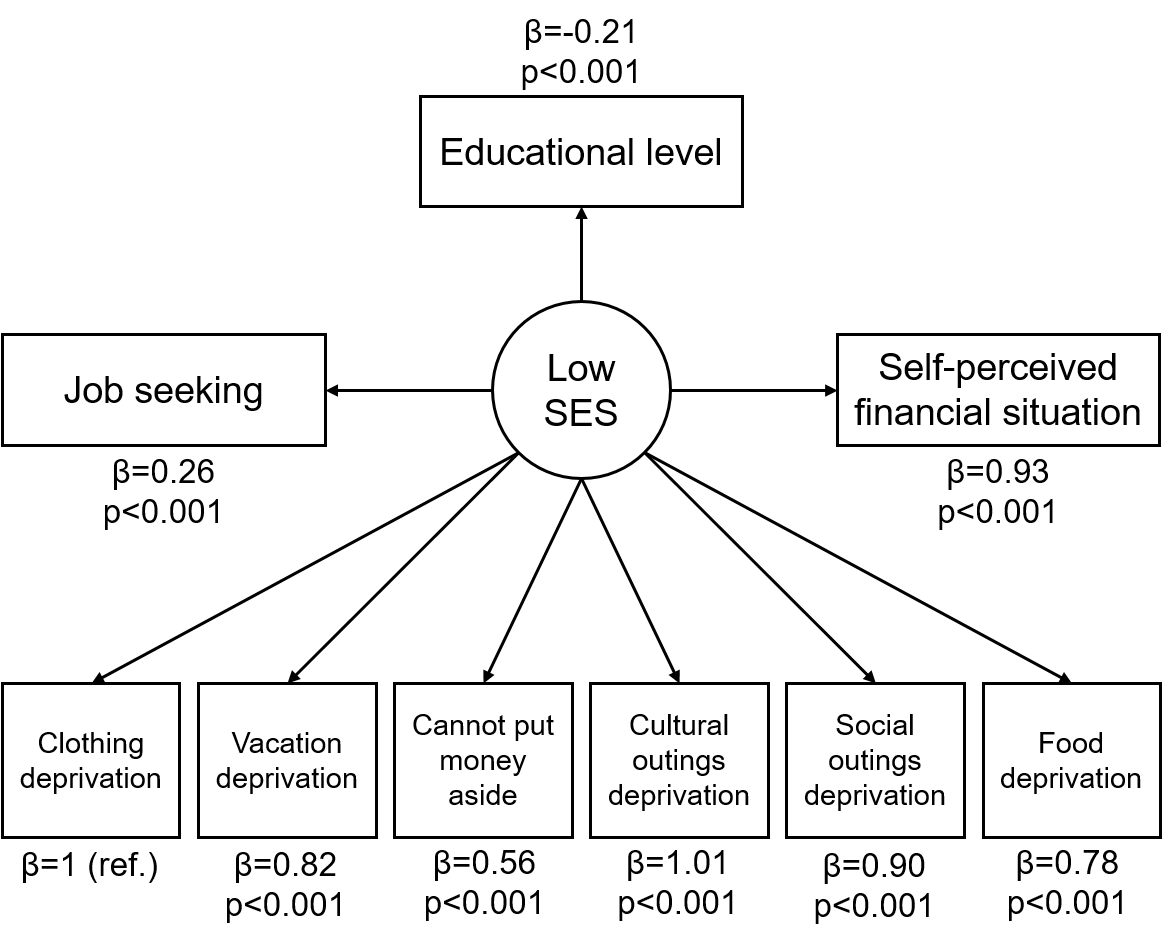

Supplement: Supplementary file 1 — Figure S1: Measurement model of the socioeconomic construct. The six squares at the bottom represent material deprivation variables. [file DAR-45-0-s005.png]
