# Supplementary material for: Crossed Pathways: Tobacco–Cannabis Co‐Use and Motivation to Quit in Young Adults in France
Source: Drug Alcohol Rev. 2026 Jun 25;45(5):e70195. doi: 10.1111/dar.70195 (PMC13305342; doi:10.1111/dar.70195)
Supplement: Supplementary file 2 — Appendix S1: Rationale for pathway design and generalised structural equation modelling hypotheses. [file DAR-45-0-s002.docx]

# Supplementary Material 1: Rationale for pathway design and generalized structural equation modelling hypotheses

## High frequency of use and dependence as barriers to readiness to quit

In young people, readiness to quit or cut down tobacco use is consistently negatively correlated with frequency of use and nicotine dependence (1–4). The latter two are related (5), on a bidirectional fashion (6–8). The same has been reported for cannabis, as quit desire was lower in young women using cannabis more frequently (9), with frequency of use being related to CUD (10), most probably with the former preceding the latter (11,12). For both substances, we therefore expected a higher frequency of use to have a negative impact on readiness to quit.

For tobacco, this frequency effect may partly be imputed to dependence, as withdrawal symptoms are seen as a barrier to quit (13–15). While this latter mechanism is less documented for cannabis (16), we made a similar assumption. However, for cannabis, experiencing cannabis-related problems or consequences, which are more likely as frequency of use increases, and which presence is associated with CUD (10), may enhance motivation to quit (9,17,18) and therefore lower the frequency-to-readiness relationship.

We may also expect a higher frequency of use to negatively impact readiness to quit independently of dependence. For instance, higher frequency of use may imply strong links to multiple daily activities and routines, and multiple learned cue–response associations that generate non-conscious impulses to act (19,20). In such a context, quitting would require great changes in routine behaviors, which may impair readiness to quit.

## Low socioeconomic status contributes to higher frequency of use and dependence

A lower SES has been associated with a higher frequency of tobacco and cannabis use (23–26). This effect may be partly explained by a higher number of friends who smoke (27,28), or no home smoking ban (29,30), which increase the opportunities to smoke. A lower SES may also enhance smoking frequency through parental smoking, norms, and early age of initiation (31–37).

Lower SES may therefore contribute to dependence by increasing frequency of use. A lower SES may thus favor dependence through higher frequencies of use. The relationship between lower SES and higher odds of nicotine dependence may also be mediated by stress and the search for alleged tobacco-related anxiolytic effects (38,39). The relationship between a lower SES and CUD (40) may also partly be explained by coping motives for cannabis use (41).

## Low socioeconomic status may also contribute to higher readiness to quit

The relationship between SES and readiness to quit is expected to be partly mediated by frequency of use and dependence. Beside those paths, a lower SES may increase readiness to quit tobacco through more stringent financial strain (42–44) or health issues (43). While it is less documented, we may expect similar SES effects on readiness to quit cannabis (45).

## More frequent use of one substance may increase readiness to quit the other

Little is known on the relationships between tobacco (respectively cannabis) use frequency and motivation to quit cannabis (respectively tobacco). A few studies reported no correlations (46) or negative ones (47), but did not investigate direct and indirect effects of frequency of use of one substance on motivation to quit the other. We may expect that both substances partly substitute for one another (48), and therefore that if one is used at high frequency, people may tend to be ready to stop the other substance. Both substances may also be perceived as depleting shared resources such as time, money, health, or tolerance of close contacts. If the use of one substance becomes dominant, it may encourage individuals to reduce or stop using the other. Lastly, as perceived loss of control may foster motivation to quit (49), we can expect that such a feeling related to a substance used at a high frequency may tend to be compensated if the other substance is quit.

## Positive correlation between tobacco and cannabis frequency of use

Tobacco and cannabis frequency of use are expected to be positively associated in people who co-use, in a bidirectional fashion, because of common routes of administration, mulling, common triggers to use, common genetic liability, and increased sensitivity to nicotine by cannabis use (48,50,51).

## Positive correlation between both levels of readiness to quit

Little is known on the relationships between both levels of readiness to quit in people who co-use (52). However, we may expect both levels to be correlated because of common enhancing exogenous factors not previously mentioned, such as social pressure and support (53,54).

# References

1. Chung T, Maisto SA, Mihalo A, Martin CS, Cornelius JR, Clark DB. Brief Assessment of Readiness to Change Tobacco Use in Treated Youth. J Subst Abuse Treat. 2011;41:137–147.

2. Prokhorov AV, Hudmon KS, de Moor CA, Kelder SH, Conroy JL, Ordway N. Nicotine dependence, withdrawal symptoms, and adolescents’ readiness to quit smoking. Nicotine Tob Res. 2001;3:151–155.

3. Berg CJ, Ling PM, Hayes RB, Berg E, Nollen N, Nehl E, et al. Smoking frequency among current college student smokers: distinguishing characteristics and factors related to readiness to quit smoking. Health Educ Res. 2012;27:141–150.

4. Masters MN, Haardörfer R, Windle M, Berg C. Psychosocial and cessation-related differences between tobacco-marijuana co-users and single product users in a college student population. Addict Behav. 2018;77:21–27.

5. Oliver JA, Foulds J. Association Between Cigarette Smoking Frequency and Tobacco Use Disorder in U.S. Adults. Am J Prev Med. 2021;60:726–728.

6. Doubeni CA, Reed G, DiFranza JR. Early Course of Nicotine Dependence in Adolescent Smokers. Pediatrics. 2010;125:1127–1133.

7. Vsevolozhskaya OA, Anthony JC. Inter-relationships Linking Probability of Becoming a Case of Nicotine Dependence With Frequency of Tobacco Cigarette Smoking. Nicotine Tob Res. 2016;18:2278–2282.

8. Song TM, An J-Y, Hayman LL, Kim GS, Lee JY, Jang HL. A Three-Year Autoregressive Cross-Lagged Panel Analysis on Nicotine Dependence and Average Smoking. Healthc Inform Res. 2012;18:115–124.

9. Caviness CM, Hagerty CE, Anderson BJ, de Dios MA, Hayaki J, Herman D, et al. Self-efficacy and motivation to quit marijuana use among young women. Am J Addict. 2013;22:373–380.

10. Callaghan RC, Sanches M, Kish SJ. Quantity and frequency of cannabis use in relation to cannabis-use disorder and cannabis-related problems. Drug Alcohol Depend. 2020;217:108271.

11. Connor JP, Stjepanović D, Le Foll B, Hoch E, Budney AJ, Hall WD. Cannabis use and cannabis use disorder. Nat Rev Dis Primers. 2021;7:1–24.

12. Nocon A, Wittchen H-U, Pfister H, Zimmermann P, Lieb R. Dependence symptoms in young cannabis users? A prospective epidemiological study. Journal of Psychiatric Research. 2006;40:394–403.

13. Marzo RR, El-Fass KA, Osman NA, Kyaw TM, Arivanandan PKA, Morgan LM, et al. Identifying the barriers of smoking cessation and predictors of nicotine dependence among adult Malaysian smokers: A cross-sectional study. Tob Induc Dis. 2022;20:109.

14. Hoeppner BB, Hoeppner SS, Carlon HA, Abry A, Darville A, Rohsenow DJ. Preparing for the Quit Day: Comparing Beliefs of Nondaily Versus Daily Young Adult Smokers as They Prepare for a Quit Attempt. Nicotine Tob Res. 2020;23:1038–1046.

15. Villanti AC, Bover Manderski MT, Gundersen DA, Steinberg MB, Delnevo CD. Reasons to quit and barriers to quitting smoking in US young adults. Fam Pract. 2016;33:133–139.

16. Liu J, Knoll SJ, Pascale MP, Gray CA, Bodolay A, Potter KW, et al. Intention to quit or reduce e-cigarettes, cannabis, and their co-use among a school-based sample of adolescents. Addict Behav. 2024;157:108101.

17. Stone BM, Gilbert DG. Validation of the Reasons for Quitting Smoking Cannabis Scale Among People with Cannabis Use Disorder. Subst Use Misuse. 2024;59:1950–1961.

18. Annaheim B, Rehm J, Neuenschwander M, Gmel G. [Quitting hash. The readiness for behaviour change among cannabis users in Switzerland]. Int J Public Health. 2007;52:233–241.

19. Rosel JF, Elipe-Miravet M, Elósegui E, Flor-Arasil P, Machancoses FH, Pallarés J, et al. Pooled Time Series Modeling Reveals Smoking Habit Memory Pattern. Front. Psychiatry [Internet]. 2020 [cited 2025 Aug 13];11. Available from: https://www.frontiersin.org/journals/psychiatry/articles/10.3389/fpsyt.2020.00049/full

20. Gardner B, Arden MA, Brown D, Eves FF, Green J, Hamilton K, et al. Developing habit-based health behaviour change interventions: twenty-one questions to guide future research. Psychology & Health. 2023;38:518–540.

21. Antonoplis S. Studying Socioeconomic Status: Conceptual Problems and an Alternative Path Forward. Perspect Psychol Sci. 2023;18:275–292.

22. Martinez SA, Beebe LA, Thompson DM, Wagener TL, Terrell DR, Campbell JE. A structural equation modeling approach to understanding pathways that connect socioeconomic status and smoking. PLoS One. 2018;13:e0192451.

23. Jeffers AM, Glantz S, Byers A, Keyhani S. Sociodemographic Characteristics Associated With and Prevalence and Frequency of Cannabis Use Among Adults in the US. JAMA Netw Open. 2021;4:e2136571.

24. Margerison-Zilko C, Cubbin C. Socioeconomic disparities in tobacco-related health outcomes across racial/ethnic groups in the United States: National Health Interview Survey 2010. Nicotine Tob Res. 2013;15:1161–1165.

25. Reid JL, Hammond D, Driezen P. Socio-economic status and smoking in Canada, 1999-2006: has there been any progress on disparities in tobacco use? Can J Public Health. 2010;101:73–78.

26. Legleye S, Beck F, Khlat M, Peretti-Watel P, Chau N. The influence of socioeconomic status on cannabis use among French adolescents. J Adolesc Health. 2012;50:395–402.

27. Hitchman SC, Fong GT, Zanna MP, Thrasher JF, Chung-Hall J, Siahpush M. Socioeconomic Status and Smokers’ Number of Smoking Friends: Findings from the International Tobacco Control (ITC) Four Country Survey. Drug Alcohol Depend. 2014;143:158–166.

28. Dias PC, Lopes S, García del Castillo JA. Tell me who your friends are?! The mediating role of friends’ use in cannabis abuse. Trends Psychiatry Psychother. 2021;44:e20200269.

29. Odo DB, Ayo-Yusuf O, Dinku Y, Mekonnen AG, Maddox R. Trends in the prevalence and factors associated with indoor smoking in 24 countries Party to the WHO FCTC: implications for equitable policy implementation. BMJ Glob Health. 2025;10:e017110.

30. Kim B, Lee Y, Kwon YD, Kim TH, Noh JW. Factors associated with indoor smoking at home by adults across Korea: a focus on socioeconomic status. Epidemiol Health. 2020;42:e2020067.

31. Dadras O, Abio A. Predictors of cigarette smoking frequency among European adolescents aged 13-15: the critical role of parental smoking and age of initiation. Eur Child Adolesc Psychiatry. 2025;

32. Hayatbakhsh MR, Najman JM, Bor W, O’Callaghan MJ, Williams GM. Multiple risk factor model predicting cannabis use and use disorders: a longitudinal study. Am J Drug Alcohol Abuse. 2009;35:399–407.

33. Phares BA, Driskill CN, Basani H, Rodil ARM, Barr EA. Multilevel Risk and Protective Factors Influencing Cannabis Use Among Adolescents and Young Adults in the United States: A Systematic Review. Compr Child Adolesc Nurs. 2025;1–29.

34. Madras BK, Han B, Compton WM, Jones CM, Lopez EI, McCance-Katz EF. Associations of Parental Marijuana Use With Offspring Marijuana, Tobacco, and Alcohol Use and Opioid Misuse. JAMA Netw Open. 2019;2:e1916015.

35. Gripe I, Danielsson A-K, Karlsson P, Thor S, Ramstedt M. Are the well-off youth in Sweden more likely to use cannabis? Drug and Alcohol Review. 2021;40:126–134.

36. O’Loughlin JL, Dugas EN, O’Loughlin EK, Winickoff JP, Montreuil A, Wellman RJ, et al. Parental Cannabis Use Is Associated with Cannabis Initiation and Use in Offspring. The Journal of Pediatrics. 2019;206:142-147.e1.

37. Hopfer CJ, Stallings MC, Hewitt JK, Crowley TJ. Family transmission of marijuana use, abuse, and dependence. J Am Acad Child Adolesc Psychiatry. 2003;42:834–841.

38. Siahpush M, McNeill A, Borland R, Fong GT. Socioeconomic variations in nicotine dependence, self‐efficacy, and intention to quit across four countries: findings from the International Tobacco Control (ITC) Four Country Survey. Tob Control. 2006;15:iii71–iii75.

39. Sim YS, Yoo S, Lee K-S, Rhee CK, Kim YK. Associations of clinical, psychological, and socioeconomic characteristics with nicotine dependence in smokers. Sci Rep. 2021;11:18544.

40. Rabiee R, Lundin A, Agardh E, Allebeck P, Danielsson A-K. Cannabis use disorder in relation to socioeconomic factors and psychiatric comorbidity: A cluster analysis of three million individuals born in 1970–2000. Scand J Public Health. 2023;51:82–89.

41. van der Pol P, Liebregts N, de Graaf R, Korf DJ, van den Brink W, van Laar M. Predicting the transition from frequent cannabis use to cannabis dependence: a three-year prospective study. Drug Alcohol Depend. 2013;133:352–359.

42. Guignard R, Soullier N, Pasquereau A, Andler R, Beck F, Nguyen-Thanh V. Factors associated with smokers’ desire to quit and quit attempts. Results from the Santé publique France Health Barometer, 2021. Bull Epidémiol Hebd. 2023;159–65.

43. Pisinger C, Aadahl M, Toft U, Jørgensen T. Motives to quit smoking and reasons to relapse differ by socioeconomic status. Preventive Medicine. 2011;52:48–52.

44. Siahpush M, Farazi PA, Maloney SI, Dinkel D, Nguyen MN, Singh GK. Socioeconomic status and cigarette expenditure among US households: results from 2010 to 2015 Consumer Expenditure Survey. BMJ Open. 2018;8:e020571.

45. Chauchard E, Septfons A, Chabrol H. [Motivations for cannabis cessation, coping and adaptation strategies, and perceived benefits: impact on cannabis use relapse and abstinence]. Encephale. 2013;39:385–392.

46. Gravely S, Driezen P, Shahab L, McClure EA, Hyland A, Cummings KM, et al. Associations of Cannabis Use, High-Risk Alcohol Use, and Depressive Symptomology with Motivation and Attempts to Quit Cigarette Smoking Among Adults: Findings from the 2020 ITC Four Country Smoking and Vaping Survey. Int J Ment Health Addiction [Internet]. 2023 [cited 2025 June 2];Available from: https://doi.org/10.1007/s11469-023-01214-8

47. Twyman L, Bonevski B, Paul C, Kay-Lambkin FJ, Bryant J, Oldmeadow C, et al. The association between cannabis use and motivation and intentions to quit tobacco within a sample of Australian socioeconomically disadvantaged smokers. Health Educ Res. 2016;31:771–781.

48. Lemyre A, Poliakova N, Bélanger RE. The Relationship Between Tobacco and Cannabis Use: A Review. Subst Use Misuse. 2019;54:130–145.

49. Arana-Chicas E, Cupertino AP, Goggin K, Richter KP, Harris KJ, Catley D. Stress and depression and quit attempt outcomes among unmotivated smokers. Subst Use Misuse. 2021;56:1564–1568.

50. Akbar SA, Tomko RL, Salazar CA, Squeglia LM, McClure EA. Tobacco and cannabis co-use and interrelatedness among adults. Addict Behav. 2019;90:354–361.

51. Badiani A, Boden JM, De Pirro S, Fergusson DM, Horwood LJ, Harold GT. Tobacco smoking and cannabis use in a longitudinal birth cohort: evidence of reciprocal causal relationships. Drug Alcohol Depend. 2015;150:69–76.

52. Ramo DE, Delucchi KL, Liu H, Hall SM, Prochaska JJ. Young adults who smoke cigarettes and marijuana: Analysis of thoughts and behaviors. Addictive Behaviors. 2014;39:77–84.

53. Soulakova JN, Tang C-Y, Leonardo SA, Taliaferro LA. Motivational Benefits of Social Support and Behavioural Interventions for Smoking Cessation. Journal of Smoking Cessation. 2018;13:216–226.

54. Chauchard E, Levin KH, Copersino ML, Heishman SJ, Gorelick DA. Motivations to quit cannabis use in an adult non-treatment sample: are they related to relapse? Addict Behav. 2013;38:2422–2427.
