# Supplementary material for: Crossed Pathways: Tobacco–Cannabis Co‐Use and Motivation to Quit in Young Adults in France
Source: Drug Alcohol Rev. 2026 Jun 25;45(5):e70195. doi: 10.1111/dar.70195 (PMC13305342; doi:10.1111/dar.70195)
Supplement: Supplementary file 3 — Table S1: Results of the first and alternative models (generalised structural equation modelling, n = 357). [file DAR-45-0-s003.docx]

**Supplementary Table 1. Results of the first and alternative models (generalized structural equation modelling, n=357)**

|  | **Model 1** |  | **Model 2** |  |
| --- | --- | --- | --- | --- |
|  | **Coefficient [95% CI]** | **p-value** | **Coefficient [95% CI]** | **p-value** |
| **Clothing deprivation^1^** |  |  |  |  |
| Low SES | 1 (ref.) | - | 1 (ref.) | - |
| **Vacation deprivation^1^** |  |  |  |  |
| Low SES | 0.82 [0.48 ;1.15] | <0.001 | 0.81 [0.48;1.14] | <0.001 |
| **Cannot put money aside^1^** |  |  |  |  |
| Low SES | 0.58 [0.34 ;0.81] | <0.001 | 0.58 [0.34;0.81] | <0.001 |
| **Cultural outings deprivation^1^** |  |  |  |  |
| Low SES | 1.00 [0.57 ;1.42] | <0.001 | 0.97 [0.56;1.39] | <0.001 |
| **Social outings derpivation^1^** |  |  |  |  |
| Low SES | 0.90 [0.52 ;1.28] | <0.001 | 0.90 [0.52;1.28] | <0.001 |
| **Food deprivation^1^** |  |  |  |  |
| Low SES | 0.78 [0.43 ;0.12] | <0.001 | 0.79 [0.44;1.14] | <0.001 |
| **Self-perceived financial situation** |  |  |  |  |
| Low SES | 0.87 [0.52 ;1.23] | <0.001 | 0.87 [0.51;1.22] | <0.001 |
| **Educational level** |  |  |  |  |
| Low SES | -0.21 [-0.31 ;-1.12] | <0.001 | -0.21 [-0.3;-0.12] | <0.001 |
| **Job seeking** |  |  |  |  |
| Low SES | 0.25 [0.12 ;0.38] | <0.001 | 0.25 [0.12;0.38] | <0.001 |
| **Tobacco use frequency** |  |  |  |  |
| Cannabis use frequency | 0.51 [0.23 ;0.78] | <0.001 | 0.42 [0.16;0.67] | 0.001 |
| Gender |  |  |  |  |
| Women | 0.15 [-0.29 ;0.58] | 0.511 | 0.11 [-0.3;0.52] | 0.604 |
| Other / Do not want to answer | -0.21 [-1.31;0.90] | 0.715 | 0.00 [-1.03;1.03] | 0.998 |
| Age | 0.06 [-0.00 ;0.12] | 0.056 | 0.05 [-0.01;0.11] | 0.074 |
| Low SES | 0.05 [-0.03 ;0.12] | 0.205 | 0.12 [0.04;0.19] | 0.003 |
| Nicotine dependence | 2.56 [2.02 ;3.09] | <0.001 | - |  |
| **Cannabis use frequency** |  |  |  |  |
| Tobacco use frequency | 0.47 [0.21 ;0.73] | <0.001 | 0.46 [0.20;0.72] | <0.001 |
| Gender |  |  |  |  |
| Women | -0.37 [-0.79 ;0.06] | 0.090 | -0.37 [-0.79;0.06] | 0.091 |
| Other / Do not want to answer | -0.11 [-1.15;0.93] | 0.830 | -0.11 [-1.15;0.93] | 0.840 |
| Age | 0.05 [-0.02 ;0.11] | 0.145 | 0.05 [-0.02;0.11] | 0.143 |
| Low SES | 0.10 [0.02 ;0.17] | 0.011 | 0.09 [0.02;0.17] | 0.013 |
| **Readiness to quit tobacco^2^** |  |  |  |  |
| Tobacco use frequency | -0.76 [-1.17 ;-0.34] | <0.001 | -0.99 [-1.35;-0.63] | <0.001 |
| Cannabis use frequency | 0.43 [0.07 ;0.80] | 0.020 | 0.47 [0.10;0.83] | 0.012 |
| Gender |  |  |  |  |
| Women | -0.58 [-1.16 ;-0.00] | 0.049 | -0.58 [-1.17;0.00] | 0.049 |
| Other / Do not want to answer | -1.78 [-3.24;-0.31] | 0.018 | -1.87 [-3.34;-0.40] | 0.013 |
| Age | 0.00 [-0.08 ;0.09] | 0.933 | 0.01 [-0.08;0.09] | 0.866 |
| Low SES | 0.15 [0.05 ;0.26] | 0.005 | 0.14 [0.04;0.24] | 0.008 |
| Nicotine dependence | -0.72 [-1.41 ;-0.02] | 0.043 | - |  |
| **Readiness to quit cannabis^2^** |  |  |  |  |
| Tobacco use frequency | 0.30 [-0.06 ;0.66] | 0.101 | 0.29 [-0.07;0.65] | 0.117 |
| Cannabis use frequency | -0.78 [-1.17 ;-0.40] | <0.001 | -0.78 [-1.17;-0.39] | <0.001 |
| Cannabis dependence^3^ | 0.65 [-0.04 ;1.34] | 0.063 | 0.66 [-0.03;1.35] | 0.061 |
| Gender |  |  |  |  |
| Women | 0.37 [-0.21 ;0.95] | 0.215 | 0.37 [-0.21;0.95] | 0.213 |
| Other / Do not want to answer | -0.15 [-1.62;1.32] | 0.841 | -0.14 [-1.62;1.33] | 0.848 |
| Age | -0.01 [-0.10 ;0.07] | 0.764 | -0.01 [-0.10;0.07] | 0.771 |
| Low SES | 0.13 [0.03 ;0.23] | 0.013 | 0.13 [0.03;0.23] | 0.014 |
| **Cannabis dependence**^3^ |  |  |  |  |
| Cannabis use frequency | 1.05 [0.72 ;1.39] | <0.001 | 1.05 [0.72;1.39] | <0.001 |
| Gender |  |  |  |  |
| Women | 0.09 [-0.48 ;0.65] | 0.761 | 0.09 [-0.48;0.65] | 0.762 |
| Other / Do not want to answer | 0.43 [-1.20;2.06] | 0.604 | 0.44 [-1.19;2.07] | 0.598 |
| Age | -0.04 [-0.12 ;0.04] | 0.289 | -0.04 [-0.12;0.04] | 0.289 |
| Low SES | 0.11 [0.01 ;0.21] | 0.026 | 0.11 [0.01;0.20] | 0.028 |
| **Low SES** |  |  |  |  |
| Gender |  |  |  |  |
| Women | 0.28 [-0.53 ;1.09] | 0.498 | 0.28 [-0.54;1.09] | 0.502 |
| Other / Do not want to answer | 2.12 [0.01;4.23] | 0.049 | 2.11 [-0.02;4.24] | 0.052 |
| Age | -0.01 [-0.13 ;0.10] | 0.814 | -0.01 [-0.13;0.10] | 0.805 |
|  |  |  |  |  |
| Low SES variance | 11.37 [5.90 ;21.92] |  | 11.60 [6.00 ;22.43] |  |
| Readiness to quit tobacco variance | 6.98 [6.02;8.09] |  | 7.03 [6.06;8.15] |  |
| Readiness to quit cannabis variance | 7.06 [6.09;8.18] |  | 7.06 [6.09;8.19] |  |
| Readiness levels covariance | 1.70 [0.94 ;2.46] | <0.001 | 1.63 [0.88;2.39] | <0.001 |
| **Nicotine dependence^4^** |  |  | - |  |
| Tobacco use frequency | 1.93 [1.48 ;2.38] | <0.001 |  |  |
| Gender |  |  |  |  |
| Women | 0.15 [-0.42 ;0.72] | 0.599 |  |  |
| Other / Do not want to answer | 0.89 [-0.50;2.27] | 0.211 |  |  |
| Age | -0.03 [-0.11;0.05] | 0.439 |  |  |
| Low SES | 0.10 [-0.00;0.20] | 0.054 |  |  |

CI, confidence interval ; SES, socioeconomic status

^1^ Deprivation variables

^2^ Self-reported on a scale from 1 (“Not at all”) to 10 (“100% ready”).

^3^ Cannabis Use Disorder Identification Test-Short Form ≥ 2 (1)

^4^ Fagerström Test for Nicotine Dependence ≥ 4 (2)

# References

1. Bonn-Miller MO, Heinz AJ, Smith EV, Bruno R, Adamson S. Preliminary Development of a Brief Cannabis Use Disorder Screening Tool: The Cannabis Use Disorder Identification Test Short-Form. Cannabis Cannabinoid Res. 2016;1:252–261.

2. Heatherton TF, Kozlowski LT, Frecker RC, Fagerström KO. The Fagerström Test for Nicotine Dependence: a revision of the Fagerström Tolerance Questionnaire. Br J Addict. 1991;86:1119–1127.
