# Supplementary material for: Crossed Pathways: Tobacco–Cannabis Co‐Use and Motivation to Quit in Young Adults in France
Source: Drug Alcohol Rev. 2026 Jun 25;45(5):e70195. doi: 10.1111/dar.70195 (PMC13305342; doi:10.1111/dar.70195)
Supplement: Supplementary file 4 — Table S2: Information criteria and model comparison metrics (generalised structural equation modelling, n = 357). [file DAR-45-0-s006.docx]

**Supplementary Table 2. Information criteria and model comparison metrics (generalized structural equation modelling, n=357)**

|  | **Log-likelihood** | **Akaike Information Criterion** | **Bayesian Information Criterion** |
| --- | --- | --- | --- |
| Model 1 | -4833.7 | 9817.4 | 10108.2 |
| Model 2 | -4728.8 | 9591.6 | 9851.4 |
