# Supplementary material for: Crossed Pathways: Tobacco–Cannabis Co‐Use and Motivation to Quit in Young Adults in France
Source: Drug Alcohol Rev. 2026 Jun 25;45(5):e70195. doi: 10.1111/dar.70195 (PMC13305342; doi:10.1111/dar.70195)
Supplement: Supplementary file 5 — Table S3: Associations between socioeconomic‐related variables and levels of readiness to quit tobacco and cannabis (linear regression models, n = 357). [file DAR-45-0-s004.docx]

**Supplementary Table 3. Associations between socioeconomic-related variables and levels of readiness to quit tobacco and cannabis (linear regression models, n=357)**

|  | **Readiness to quit tobacco** |  | **Readiness to quit cannabis** |  |
| --- | --- | --- | --- | --- |
|  | **Coef. [95%CI]** | **p-value** | **Coef. [95%CI]** | **p-value** |
| **Educational level^1^** | -0.05 [-0.21;0.11] | 0.531 | -0.06 [-0.23;0.10] | 0.438 |
| **Job seeking** | 0.42 [-0.31;1.15] | 0.260 | 0.19 [-0.56;0.93] | 0.620 |
| **Self-perceived financial situation** |  | 0.649 |  | 0.187 |
| You are rather or really comfortable (ref.) | 1 |  | 1 |  |
| You are ok | 0.19 [-0.69;1.07] | 0.666 | 0.61 [-0.28;1.51] | 0.178 |
| You just get by | 0.15 [-0.68;0.99] | 0.722 | 0.95 [0.10;1.79] | 0.029 |
| It’s difficult to make ends meet / You can’t manage without going into debt | 0.51 [-0.33;1.34] | 0.235 | 0.58 [-0.27;1.43] | 0.183 |
| **Material deprivation** |  |  |  |  |
| You often have to go without clothing for financial reasons | 0.72 [0.03;1.41] | 0.040 | 0.71 [0.01;1.41] | 0.048 |
| You often have to go without trips or holidays for financial reasons | 0.46 [-0.12;1.04] | 0.120 | 0.59 [-0.01;1.18] | 0.052 |
| You cannot put money aside at the end of the month | 0.82 [0.23;1.41] | 0.007 | 0.88 [0.28;1.48] | 0.004 |
| You often have to go without cultural outings for financial reasons | 0.54 [-0.13;1.21] | 0.111 | 0.40 [-0.29;1.08] | 0.256 |
| You often have to go without going out to bars, restaurants, or nightclubs for financial reasons | 0.73 [0.10;1.36] | 0.024 | 0.59 [-0.06;1.23] | 0.075 |
| You often have to go without food for financial reasons | 0.87 [0.01;1.73] | 0.047 | 0.49 [-0.39;1.37] | 0.274 |
| **Composite material deprivation^2^** | 0.22 [0.06;0.37] | 0.005 | 0.20 [0.05;0.35] | 0.011 |

CI, confidence interval.

For each variable, a separate model was run. Models were adjusted for frequency of tobacco and cannabis use, cannabis dependence, age, and gender.

^1^ This variable, ranging from 0 to 8, corresponded to the highest diploma obtained in the following list: Lower secondary school diploma (*Brevet des collèges*); Vocational diploma (*BEP, CAP, BP, etc.*); Vocational baccalaureate; General or technological baccalaureate; Two-year higher education diploma (*BTS, DUT, etc.*); Three- or four-year higher education degree (*Licence, Maîtrise, etc*.); Master’s degree or higher (Master, engineering degree, etc.).

^2^ This variable, ranging from 0 to 6, corresponds to the sum of self-reported deprivations.
