# Supplementary material for: Crossed Pathways: Tobacco–Cannabis Co‐Use and Motivation to Quit in Young Adults in France
Source: Drug Alcohol Rev. 2026 Jun 25;45(5):e70195. doi: 10.1111/dar.70195 (PMC13305342; doi:10.1111/dar.70195)
Supplement: Supplementary file 6 — Table S4: Factors associated with having made at least 3 lifetime cessation attempts (binary logistic regressions, n = 357). [file DAR-45-0-s001.docx]

**Supplementary Table 4. Factors associated with having made at least 3 lifetime cessation attempts (binary logistic regressions, n=357)**

|  | **Tobacco** |  | **Cannabis** |  |
| --- | --- | --- | --- | --- |
|  | **aOR [95% CI]** | **p-value** | **aOR [95% CI]** | **p-value** |
| **Age, in years (median [IQR])** | 1.05 [0.98;1.12] | 0.193 | 1.06 [0.99;1.13] | 0.108 |
| **Gender** |  |  |  |  |
| Men (ref.) | 1 |  | 1 |  |
| Women | 0.62 [0.39;1.00] | 0.052 | 0.80 [0.50;1.26] | 0.335 |
| Other / Do not want to answer | 0.51 [0.16;1.65] | 0.261 | 1.78 [0.49;6.41] | 0.377 |
| **Composite material deprivation^1^** | 1.27 [1.13;1.43] | <0.001 | 1.11 [0.99;1.25] | 0.080 |
| **Educational level^2^** | 1.08 [0.95;1.23] | 0.221 | 1.00 [0.88;1.13] | 0.942 |
| **Tobacco use frequency** |  |  |  |  |
| Non-daily use (ref.) | 1 |  | 1 |  |
| < 10 cigarettes per day | 0.36 [0.19;0.66] | 0.001 | 0.59 [0.32;1.07] | 0.081 |
| ≥ 10 cigarettes per day | 0.42 [0.23;0.76] | 0.004 | 0.90 [0.51;1.60] | 0.720 |
| **Cannabis use frequency** |  |  |  |  |
| < 10 days per month (ref.) | 1 |  | 1 |  |
| 10-29 days per month | 0.79 [0.41;1.54] | 0.489 | 1.40 [0.75;2.62] | 0.294 |
| Daily use | 1.45 [0.76;2.75] | 0.257 | 1.94 [1.04;3.63] | 0.037 |

CI, confidence interval; IQR, interquartile range; RRR, relative risk ratio.

^1^ This variable, ranging from 0 to 6, corresponds to the sum of self-reported deprivations.

^2^ This variable, ranging from 0 to 8, corresponded to the highest diploma obtained in the following list: Lower secondary school diploma (*Brevet des collèges*); Vocational diploma (*BEP, CAP, BP, etc.*); Vocational baccalaureate; General or technological baccalaureate; Two-year higher education diploma (*BTS, DUT, etc.*); Three- or four-year higher education degree (*Licence, Maîtrise, etc*.); Master’s degree or higher (Master, engineering degree, etc.).
